# Intimate Pride: a Tri-Nation Study on Associations between Positive Minority Identity Aspects and Relationship Quality in Sexual Minorities from German-Speaking Countries

**DOI:** 10.1007/s41042-022-00070-6

**Published:** 2022-08-02

**Authors:** Magdalena Siegel, Ashley K. Randall, Pamela J. Lannutti, Melanie S. Fischer, Yuvamathi Gandhi, Raphaela Lukas, Nathalie Meuwly, Orsolya Rosta-Filep, Katharina van Stein, Beate Ditzen, Tamás Martos, Carmen Schneckenreiter, Casey J. Totenhagen, Martina Zemp

**Affiliations:** 1 Department of Clinical and Health Psychology, University of Vienna, Vienna, Austria; 2 Department of Public Health, Institute of Tropical Medicine, Antwerpen, Belgium; 3 Counseling and Counseling Psychology, Arizona State University, Tempe, USA; 4 Center for Human Sexuality Studies, Widener University, Chester, USA PA; 5 Institute of Medical Psychology, Heidelberg University Hospital & Heidelberg University, Heidelberg, Germany; 6 Department of Psychology, Institute for Family Research and Counseling, University of Fribourg, Fribourg, Switzerland; 7 Doctoral School, Semmelweis University, Budapest, Hungary; 8 Department of Personality, Clinical and Health Psychology, University of Szeged, Szeged, Hungary; 9 Human Development and Family Studies, University of Alabama, Tuscaloosa, USA AL

## Abstract

Investigations into the intimate relationships of sexual minorities are proliferating, but often adopt a deficit-oriented and US-centered perspective. In this tri-nation online study with sexual minority participants from Austria, Germany, and Switzerland (N = 571), we (i) assessed the construct validity of the German version of a well-known measure for positive minority identity aspects (the Lesbian, Gay, Bisexual Positive Identity Measure; LGB-PIM), and (ii) explored associations between these aspects (self-awareness, authenticity, community, capacity for intimacy, and social justice) and self-reported relationship quality. Model fit of the German version of the LGB-PIM was deemed acceptable. Higher levels of positive minority identity aspects showed small to moderate associations with higher levels of relationship quality in bivariate analyses, but only capacity for intimacy was linked to relationship quality in higher-order models (controlling for country, age, sexual orientation, gender identity, relationship length, and psychological distress). Results remained robust in several sensitivity analyses. Our results highlight the differential role of positive identity aspects for relationship functioning, with capacity for intimacy as a fruitful leverage point for therapeutic work.

## Background

Psychological research on the intimate relationship(s) of people who identify as a sexual minority (i.e., lesbian, gay, bisexual, and other people who do not identify as heterosexual) has proliferated in recent years (see Meuwly & Randall, 2019; Rostosky & Riggle, 2017). Much of this research has focused on the unique experiences of these individuals, particularly from a social stress perspective (LeBlanc et al., 2015; Meyer, 2003), to elucidate unique risk factors for relationship functioning in this population (Rostosky & Riggle, 2017). Less is known about how positive identity aspects related to a minoritized sexual orientation ? such as increased self-awareness and authenticity, belonging to a community, enjoying intimate relationships outside traditional norms, or advocating for social justice ? may be associated with relational outcomes. Further, most of the extant research has examined related constructs using samples from the United States (US), which limits a global understanding. Thus, we explore patterns of associations between positive sexual minority identity aspects and relationship quality in sexual minorities from three German-speaking countries, specifically Austria, Germany, and Switzerland.

### The Intimate Relationships of Sexual Minorities: Beyond a Deficit Orientation

Early research on the relationship experiences of sexual minorities typically contrasted (i) heterosexually-identified individuals with sexual minority individuals and/or (ii) individuals in mixed-gender relationships with individuals in same-gender relationships (Lavner, 2017). Recent research examined the relational impact of characteristics that are unique to sexual minorities, most commonly minority stress (Rostosky & Riggle, 2017). Minority stress (Meyer, 2003) refers to stress that sexual minorities experience because of their marginalized identity, including discrimination and prejudice (Meyer, 2003) or internalizations of negative societal attitudes (Berg et al., 2016). Minority stress shows robust associations with mental health concerns (e.g., Newcomb & Mustanski 2010) and has been proposed to account for the higher levels of psychological distress found in these populations (Hatzenbuehler, 2009; Meyer, 2003).

Empirical (e.g., Feinstein et al., 2019; Meuwly & Davila, 2021; Newcomb et al., 2021; Totenhagen et al., 2018) and theoretical (e.g., LeBlanc et al., 2015; Newcomb, 2020) work in this area typically integrates minority stressors into broader models of stress, coping, and relationship functioning (e.g., Karney & Bradbury 1995; Randall & Bodenmann, 2017). Across this work, it is postulated (or found empirically) that minority stress has a negative association with mental health (LeBlanc et al., 2015), engagement in intimacy and public displays of affection (Guschlbauer et al., 2019; Hocker et al., 2021; Szymanski & Hilton, 2013), as well as individual and dyadic coping resources (Meuwly & Davila, 2021; Totenhagen et al., 2018), collectively demonstrating the negative associations between minority stress and relationship functioning. These findings emphasize the importance of considering correlates of relationship functioning that are unique to sexual minorities.

Minority stress research has also received scholarly criticism, including from positive psychologists (Vaughan et al., 2014). This criticism centers around the deficit-oriented perspective inherent to minority stress research that focuses on elucidating risk rather than protective factors for health and relational outcomes. Overemphasizing the link between psychopathology and sexual orientation (if not addressed carefully; see Meyer 2003) might carry the risk of further stigmatizing sexual minority individuals (Eaton et al., 2021; Frost, 2017), and has limited explanatory value as to which factors contribute to the individual and relational well-being and thriving of this population above and beyond the absence of minority stress (Frost, 2017; Hill & Gunderson, 2015; Riggle et al., 2008). Thus, from a positive psychology perspective, it is critical to identify protective factors for individual and relational well-being to foster resilience in sexual minority individuals and inform evidence-based clinical practice (Riggle et al., 2008).

### Positive Minority Identity Aspects in Sexual Minorities

Sexual minorities have unique positive experiences that may shape the development, initiation, and maintenance of their romantic relationships (Meuwly & Randall, 2019). Early qualitative work conducted with samples from the US (Riggle et al., 2008; Rostosky et al., 2010) found a range of distinct positive sexual minority identity aspects relating to intrapersonal (e.g., increased empathy and compassion, authenticity, personal insight) and relational domains (e.g., belonging to a community, creating families of choice, exploring sexuality and relationships, engaging in activism). Similar themes were also found in a sample of sexual minorities from 15 Spanish-speaking countries in the Americas and Europe; Almario et al., 2013).

Subsequent quantitative work leveraged these accounts psychometrically by creating a multifactor positive identity self-report measure (the Lesbian, Gay, and Bisexual Positive Identity Measure [LGB-PIM]; Riggle et al., 2014) with the following five distinct aspects of a positive sexual minority identity (henceforth shortened to positive minority identity): (1) *self-awareness* (i.e., believing that one’s sexual identity has increased one’s self-awareness), (2) *authenticity* (i.e., comfort with one’s own identity and its expression), (3) *community* (i.e., a sense of involvement with and support from the LGBTIQA+ [lesbian, gay, bisexual, transgender, intersex, queer, asexual, and other sexual orientations, gender identities, and sex characteristics] community), (4) *intimacy* (i.e., believing that one’s sexual identity has enhanced the capacity for intimacy and sexual freedom), and (5) *social justice* (i.e., believing that one’s sexual identity has increased one’s concern for social justice; all definitions abbreviated from Riggle et al., 2014). Associations between positive minority identity aspects and mental health as well as their incremental validity over minority stress have received empirical support, particularly so for positive mental health outcomes, such as life satisfaction (Riggle et al., 2014; Rostosky et al., 2018). To our knowledge, the LGB-PIM remains the only measure that comprehensively assesses several dimensions of positive minority identity formation in sexual minorities (see Mohr & Kendra 2011, for a subscale assessing identity affirmation, however).

### Positive Minority Identity Aspects and Romantic Relationship Functioning

Positive minority identity aspects seem to be promising candidates for fostering resilience and well-being in sexual minorities (Rostosky et al., 2018). Considering the inherent relational nature of some of these aspects (e.g., intimacy), it is surprising that more research has not focused on associations with relational outcomes specifically (Pepping et al., 2019). To the best of our knowledge, only one published study has linked positive minority identity aspects (as assessed by the LGB-PIM; Riggle et al., 2014) with sexual and relationship satisfaction (Mark et al., 2020). In this study on bisexual individuals in mixed-gender relationships, positive associations between the intimacy dimension of the LGB-PIM (but not other identity aspects) and sexual satisfaction (but not relationship satisfaction) were found (Mark et al., 2020). However, it is unclear to what extent these results (Mark et al., 2020) generalize to people with other non-heterosexual orientations or in other relationship constellations (e.g., same-gender couples), who are not afforded the privileges of appearing to belong to a “majority” group.

Further studies on related constructs or populations allow for tentative hypotheses regarding positive associations between the five positive minority identity aspects (i.e., self-awareness, authenticity, community, intimacy, social justice), as assessed by the LGB-PIM (Riggle et al., 2014) and positive relationship outcomes. General predecessors or correlates of self-awareness, such as self-concept clarity and emotional intelligence and awareness, have been linked to higher relationship functioning in heterosexual populations (Croyle & Waltz, 2002; Malouff et al., 2014; Parise et al., 2019), as has having a general sense of authenticity (Brunell et al., 2010). Constructs related to minority-specific authenticity, such as self-acceptance and identity affirmation (i.e., being proud of one’s sexual identity, Mohr & Kendra 2011), have been linked to higher relationship satisfaction in sexual minorities (Elizur & Mintzer, 2003; Pepping et al., 2019), but null findings exist (Vencill et al., 2018). Studies focusing on individuals identifying as Latino/a similarly point to the positive influence of having a strong ethnic identity on relational outcomes (Maiya et al., 2021; Trail et al., 2012).

Associations between intimacy and relationship quality have a strong theoretical and empirical basis (Reis & Shaver, 1988). Intimacy is typically conceptualized as a dynamic process with reciprocal emotional disclosure and responsiveness as hallmark features (Ditzen et al., 2019; Reis & Shaver, 1988) and can be understood as a component of overall relationship quality (Fletcher et al., 2000). In previous psychobiological studies on heterosexual couples, intimacy behavior (e.g., eye contact, affectionate touch) was associated with improved stress-resilience (e.g., Ditzen et al., 2007, 2019). Self-report studies with sexual minorities documented positive associations of emotional intimacy (Guschlbauer et al., 2019) and negative associations of fear of intimacy (Szymanski & Hilton, 2013) with relationship satisfaction and quality. Critically, however, intimacy as assessed by the LGB-PIM (Riggle et al., 2014) differs from prominent conceptualizations of intimacy and can be best understood as believing that one’s sexual identity has led to an increased capacity for experiencing intimacy in relationships more generally. This *capacity for intimacy* has been linked to sexual (but not relationship) satisfaction in bisexual individuals with both bisexual and straight partners, as described above (Mark et al., 2020).

The literature on community and social justice related positive minority identity aspects and relationship quality is more mixed, possibly owing to a changing sociolegal climate for sexual minorities (Haas & Lannutti, 2021; Rostosky et al., 2009). For example, higher levels of community connectedness were associated with higher levels of relationship strain in sexual minorities from New York City, US (Frost & Meyer, 2009). In another study (Haas & Lannutti, 2021), seeking out supportive environments for sexual minorities (e.g., bars) was positively related to some (relational closeness and resilience), but not all (commitment and satisfaction), assessed aspects of relationship functioning. Speculatively, there might be less need for supportive environments and community resources in a more progressive sociolegal environment (Haas & Lannutti, 2021), thus warranting further examination in regions with differing institutional support.

Social justice broadly refers to believing and advocating for equality among social groups (for a review see Louis et al., 2014). Related to this, egalitarianism has been linked to greater relationship quality in a diverse sample of individuals in interracial and/or same-gender relationships (Rosenthal & Starks, 2015). Being a social justice advocate for sexual minorities might similarly be associated with critical reflections of heterosexist societal structures and institutions, which, in turn, increases the investment or pride in one’s own relationship outside those structures. Valuing social justice might also translate into more egalitarian relationship processes (e.g., division of household labor), which has also been linked to relationship quality in same-gender couples (e.g., Sutphin 2010).

### The Need for Research in German-Speaking Countries

To date, most research on relationship functioning in sexual minorities has focused on relatively homogenous samples from the US (e.g., Cao et al., 2017; Doyle & Molix, 2015; Lavner, 2017). This is a critical limitation, as the lived experiences of sexual minorities have been shown to vary substantially regarding the sociolegal climate (i.e., laws and societal attitudes; Pachankis & Bränström 2018; Siegel et al., 2021). For partnered individuals, the influence of the sociolegal climate might be even more pronounced, as the presence or absence of institutions that provide legal security in relationships (e.g., marriage or civil unions) as well as the surrounding public discourse might be more salient to them (Lannutti, 2014; Rostosky et al., 2009).

In Austria, Germany, and Switzerland, research on sexual minorities is sparse and representative estimates on their number are lacking, despite calls for targeted, population-based, and high-quality data collection efforts (e.g., Bränström et al., 2019; Plöderl et al., 2019). A recent large-scale, European Union-wide survey among sexual and gender minorities documented high levels of minority stress for partnered individuals in these countries: 39% (Austria) and 45% (Germany) reported avoiding holding hands with their partner in public out of fear of assault and harassment (European Union Agency for Fundamental Rights, 2020). Further, full marriage equality was only recently achieved in Germany (2017), Austria (2019), and Switzerland (2021; coming into effect 2022;  Der Bundesrat, 2021); ILGA World et al., 2020), whereas previous institutions for same-gender couples (e.g., civil unions) were not equal to mixed-gender marriages in terms of spousal duties and responsibilities (ILGA World et al., 2020). The associated feeling of being a “second-class” citizen has been proposed to impact the relational and individual well-being of sexual minorities (Siegel et al., 2021).

Thus, investigations into unique correlates of relational outcomes in this population are critically needed, but are few in Switzerland (Meuwly & Davila, 2021; Meuwly et al., 2013) and currently lacking in Austria and Germany. Additionally, it is unknown to what extent relational experiences of sexual minorities in these countries are comparable to those from the US, which has important implications for global policies and clinical recommendations for these populations (e.g., American Psychological Association, APA Task Force on Psychological Practice with Sexual Minority Persons, 2021).

### The Present Study

The main aim of the present tri-nation study (Austria, Germany, Switzerland) is to assess patterns of associations between positive minority identity aspects and relationship quality in sexual minorities. To establish the validity of the German version of the LGB-PIM used in our study, we first assessed the construct validity of this measure.

The hypotheses for this study were preregistered (https://osf.io/h4dnt) and are as follows. First, we predicted that the German-version of the LGB-PIM would show acceptable model fit (Hypothesis 1). Second, we predicted positive associations between the five positive minority identity aspects (i.e., self-awareness, authenticity, community, intimacy, social justice) in sexual minorities and their self-reported relationship quality, after controlling for relevant confounds, namely age, gender identity, sexual orientation (couple gender in a sensitivity analysis), relationship length, and psychological distress (Hypothesis 2). Associations of key predictor and outcome variables with age (Bühler et al., 2021, for relationship quality; Baiocco et al., 2020, for positive identity aspects), sexual orientation or couple gender (Morandini et al., 2018, for relationship quality; Baiocco et al., 2020, for positive identity aspects), and psychological distress (Braithwaite & Holt-Lunstad, 2017, for relationship quality; Riggle et al., 2014, for positive identity aspects) have been documented in the literature. Relationship quality has also been found to be associated with relationship length (Bühler et al., 2021) and with gender identity in sexual and gender minority populations (Marshall et al., 2020; Sommantico et al., 2019, 2020; Song et al., 2021). While evidence regarding associations with positive identity aspects is (to the best of our knowledge) lacking (for relationship length) or suggests no effect (for gender identity; Petrocchi et al., 2020), we decided to include both covariates nonetheless due to their associations with relationship quality.

Because relationship quality is a multidimensional construct that shows differential associations with external variables on the subscale level (e.g., Hassebrauck & Fehr 2002; Siffert & Bodenmann, 2010), supplementary analyses were conducted to examine the associations between the five positive minority identity aspects and subdimensions of relationship quality (exploratory; no hypotheses specified).

## Method

### Open Science Practices

The hypotheses and analytic strategy (including R code) were preregistered after data collection but before conducting any inferential analyses (https://osf.io/h4dnt). The preregistration outlined an analysis plan that included cross-cultural comparisons between German-speaking countries and the US, as this study is part of a larger, international project (see below). After preregistration, cases of possibly spurious responding in the US dataset were detected. The lead authors [MS, AKR, PJL, MZ] decided to exclude the US dataset from further analyses for this manuscript (see OSF-Supplement S1 for detailed reasoning and any other deviations from protocol). R code necessary to reproduce all analyses, tables, as well as datasets and supplementary materials S1 to S7 are provided at https://osf.io/94k8x.

### Overarching Research Project and Inclusion Criteria

Data for the current study were collected as part of a larger research project on the lived experiences of sexual and gender minorities around the world (PIs: [AKR, PJL]; https://osf.io/tsj8v). Eligibility criteria included (i) being at least 18 years old, (ii) identifying as a sexual (i.e., lesbian, gay, bisexual, pansexual, queer, or otherwise non-heterosexual) and/or gender minority (i.e., transgender, non-binary, or otherwise non-cisgender), and (iii) residence in any of the participating countries. Participants were further excluded based on very short (< 600 s) or long (> 24 h) survey completion times. For the current analyses, the sample was restricted to participants who (i) lived in Austria, Germany, or Switzerland, (ii) did not identify as heterosexual, asexual, or demisexual (i.e., experiencing sexual attraction only after forming an emotional connection), and (iii) indicated being in a relationship with one or more people at time of data collection. Asexual (*n* = 11) and demisexual (*n* = 8) participants were excluded due to low case numbers and conceptual reasons preventing collapsing with other sexual orientation groups (Timmins et al., 2021).

### Participant Characteristics

Main participant characteristics by country are reported in Tables 1 and 2. The final sample comprised 571 participants (Austria: *n* = 138; Germany: *n* = 346; Switzerland: *n* = 87), who defined their sexual orientation predominantly as lesbian/gay (59%) and their gender identity as cis-female (56%), and who held a university degree (54%). 59% reported being in a same-gender relationship, with an average relationship length of *M* = 7.8 years (*SD* = 7.9; range = 0.1 to 47.2 years). Thirty-eight participants (7%) reported that their relationship was polyamorous.

**Table 1:** Sample Descriptives (Overall and by Country)

Characteristic	Overall	By Country				
	*N* = 571	Austria, *n* = 138	Germany, *n* = 346	Switzerland, *n* = 87	*p*-value^a^	Effect size^b^
Age (Years)	34.53 (11.72)	36.06 (11.66)	34.54 (11.78)	31.99 (11.28)	0.048	0.01
Missing Values	45	6	33	6		
Education					< 0.001	0.20
Compulsory Education	12 (2.1%)	3 (2.2%)	3 (0.9%)	6 (6.9%)		
National Vocational Qualification	64 (11%)	6 (4.3%)	34 (9.8%)	24 (28%)		
High-School Or Nursing Diploma	167 (29%)	33 (24%)	109 (32%)	25 (29%)		
University Degree	306 (54%)	86 (62%)	190 (55%)	30 (34%)		
Other	22 (3.9%)	10 (7.2%)	10 (2.9%)	2 (2.3%)		
Sexual Orientation					0.335	0.02
Lesbian/Gay	338 (59%)	91 (66%)	194 (56%)	53 (61%)		
Bi/Pluri	153 (27%)	31 (22%)	98 (28%)	24 (28%)		
Queer/Other	80 (14%)	16 (12%)	54 (16%)	10 (11%)		
Gender Identity					0.017	0.08
Cis-Male	81 (14%)	29 (21%)	36 (10%)	16 (18%)		
Cis-Female	321 (56%)	77 (56%)	198 (57%)	46 (53%)		
Gender-Minority	168 (29%)	32 (23%)	111 (32%)	25 (29%)		
Missing Values	1	0	1	0		
Couple Gender					0.039	0.07
Same-Gender-Couple	338 (59%)	96 (70%)	187 (54%)	55 (63%)		
Mixed-Gender-Couple	42 (7.4%)	8 (5.8%)	28 (8.1%)	6 (6.9%)		
Gender-Minority-Couple	189 (33%)	34 (25%)	129 (38%)	26 (30%)		
Missing Values	2	0	2	0		
Psychological Distress (DASS-21)	0.71 (0.64)	0.63 (0.62)	0.73 (0.64)	0.76 (0.65)	0.201	< 0.01
Missing Values	2	0	1	1		
Relationship Length (Months)	93.90 (95.09)	93.25 (82.06)	98.81 (101.47)	73.88 (85.56)	0.126	< 0.01
Missing Values	52	11	28	13		
Self-Awareness	5.75 (0.97)	5.98 (0.84)	5.68 (1.02)	5.64 (0.91)	0.007	0.02
Missing Values	26	3	19	4		
Authenticity	6.18 (0.94)	6.45 (0.74)	6.11 (0.97)	6.04 (1.01)	< 0.001	0.03
Community	5.16 (1.37)	5.22 (1.40)	5.06 (1.38)	5.47 (1.25)	0.043	0.01
Missing Values	34	6	26	2		
Intimacy	5.34 (1.32)	5.39 (1.34)	5.30 (1.31)	5.45 (1.35)	0.585	< 0.01
Missing Values	64	10	46	8		
Social Justice	6.17 (0.90)	6.18 (0.95)	6.17 (0.87)	6.13 (0.92)	0.919	< 0.01
Missing Values	13	3	10	0		
Relationship Quality (PRQC)	6.03 (0.78)	6.10 (0.83)	5.97 (0.78)	6.14 (0.68)	0.091	< 0.01

*Note*. *M (SD)* are reported for continuous variables, and *n* (%) for categorical variables. ^a^ One-Way ANOVA or Pearson’s chi-squared test (expected cell counts ≥ 5) or Fisher’s exact test (expected cell counts < 5); ^b^ Adj. Cramér’s V (*φ*_*c*_ ; cat.) or η^2^ (cont.)

**Table 2:** Sample Descriptives by Sexual Orientation and Gender Identity

Characteristic	Sexual Orientation					Gender Identity				
	Lesbian/Gay, *n* = 338	Bi/Pluri, *n* = 153	Queer/Other, *n* = 80	*p*-value^a^	Effect Size^b^	Cis-Male, *n* = 81	Cis-Female, *n* = 321	Gender-Minority, *n* = 168	*p*-value^a^	Effect Size^b^
Age (Years)	37.57 (12.31)	31.18 (10.07)	28.28 (7.01)	< 0.001	0.10	41.80 (14.66)	34.00 (10.29)	32.01 (11.32)	< 0.001	0.07
Missing Values	28	12	5			6	31	8		
Education				0.052	0.07				0.113	0.07
Compulsory Education	7 (2.1%)	3 (2.0%)	2 (2.5%)			0 (0%)	5 (1.6%)	7 (4.2%)		
National Vocational Qualification	49 (14%)	13 (8.5%)	2 (2.5%)			5 (6.2%)	40 (12%)	19 (11%)		
High-School Or Nursing Diploma	90 (27%)	47 (31%)	30 (38%)			21 (26%)	98 (31%)	48 (29%)		
University Degree	180 (53%)	82 (54%)	44 (55%)			54 (67%)	166 (52%)	85 (51%)		
Other	12 (3.6%)	8 (5.2%)	2 (2.5%)			1 (1.2%)	12 (3.7%)	9 (5.4%)		
Sexual Orientation									< 0.001	0.29
Lesbian/Gay						69 (85%)	218 (68%)	50 (30%)		
Bi/Pluri						8 (9.9%)	77 (24%)	68 (40%)		
Queer/Other						4 (4.9%)	26 (8.1%)	50 (30%)		
Gender Identity				< 0.001	0.29					
Cis-Male	69 (20%)	8 (5.2%)	4 (5.0%)							
Cis-Female	218 (65%)	77 (50%)	26 (32%)							
Gender-Minority	50 (15%)	68 (44%)	50 (62%)							
Missing Values	1	0	0							
Couple Gender				< 0.001	0.46				< 0.001	0.65
Same-Gender-Couple	281 (83%)	37 (24%)	20 (25%)			73 (91%)	265 (83%)	0 (0%)		
Mixed-Gender-Couple	1 (0.3%)	37 (24%)	4 (5.0%)			5 (6.2%)	37 (12%)	0 (0%)		
Gender-Minority-Couple	55 (16%)	78 (51%)	56 (70%)			2 (2.5%)	19 (5.9%)	168 (100%)		
Missing Values	1	1	0			1	0	0		
Psychological Distress (DASS-21)	0.56 (0.56)	0.90 (0.70)	0.98 (0.65)	< 0.001	0.08	0.42 (0.45)	0.63 (0.58)	1.01 (0.71)	< 0.001	0.10
Missing Values	1	1	0			0	1	1		
Relationship Length (Months)	108.11 (103.65)	80.58 (83.13)	56.29 (55.87)	< 0.001	0.04	118.92 (96.38)	86.96 (80.94)	94.46 (115.80)	0.030	0.01
Missing Values	27	13	12			2	33	17		
Self-Awareness	5.76 (0.97)	5.66 (1.02)	5.89 (0.87)	0.243	< 0.01	5.66 (0.99)	5.65 (1.01)	5.97 (0.86)	0.002	0.02
Missing Values	17	7	2			1	20	5		
Authenticity	6.38 (0.79)	5.92 (1.09)	5.87 (1.00)	< 0.001	0.06	6.29 (0.88)	6.30 (0.88)	5.90 (1.02)	< 0.001	0.04
Community	5.28 (1.33)	4.80 (1.49)	5.36 (1.16)	< 0.001	0.03	4.84 (1.57)	5.22 (1.32)	5.21 (1.35)	0.078	< 0.01
Missing Values	20	11	3			2	27	5		
Intimacy	5.49 (1.26)	5.05 (1.32)	5.27 (1.49)	0.004	0.02	5.36 (1.24)	5.48 (1.27)	5.09 (1.42)	0.015	0.02
Missing Values	38	19	7			6	44	14		
Social Justice	6.08 (0.93)	6.22 (0.91)	6.42 (0.66)	0.008	0.02	5.99 (0.97)	6.15 (0.91)	6.27 (0.83)	0.076	< 0.01
Missing Values	10	2	1			3	6	4		
Relationship Quality (PRQC)	6.09 (0.78)	6.02 (0.74)	5.78 (0.83)	0.006	0.02	5.80 (0.75)	6.17 (0.69)	5.86 (0.90)	< 0.001	0.05

*Note*. *M (SD)* are reported for continuous variables, and *n* (%) for categorical variables. ^a^ One-Way ANOVA or Pearson’s chi-squared test (expected cell counts ≥ 5) or Fisher’s exact test (expected cell counts < 5); ^b^ Adj. Cramér’s V (*φ*_*c*_ ; cat.) or η^2^ (cont.) One participant had a missing value for gender identity.

Across countries, participants differed significantly in average age, education level, gender identity, and couple gender, but not regarding sexual orientation and average relationship length. Excepting education (φ_c_ = 0.20), the significant differences were trivial to small in effect strength (η^2^ = 0.01 for age, φ_c_ = 0.08 for gender identity, φ_c_ = 0.07 for couple gender).

### Sampling Procedure

All study materials and procedures were approved by respective institutional review boards prior to data collection (Austria: University of Vienna, reference number: 00702, date of approval: July 9, 2021; Germany: Heidelberg University Hospital & Heidelberg University, ZB 46221, June 29, 2021; Switzerland: University of Fribourg, 2021−705, June 24, 2021). Data were collected online from July 14, 2021 to October 13, 2021 using the platform SoSci Survey. Participants were recruited primarily via organizations for sexual and gender minorities that served as multipliers for disseminating recruitment materials provided by the research teams (see preregistration for details). Participants could participate in a gift raffle with prizes of varying amounts according to study site (Austria: 2 × 200 €; 2 × 50 €; 16 × 20 €; Germany: 10 × 50 €; Switzerland: 5 × 50 CHF). E-mail addresses were stored separately from study data and no IP addresses were collected. Participation was anonymous and voluntary. Informed consent was obtained after initial screening questions determining eligibility.

### Measures

#### Positive Minority Identity Aspects

Positive minority identity aspects were assessed using the LGB-PIM (Riggle et al., 2014). The LGB-PIM assesses five positive minority identity aspects: *self-awareness* (e.g., “My LGBT identity leads me to important insights about myself”), *authenticity* (e.g., “I feel I can be honest and share my LGBT identity with others”), *community* (e.g., “I feel a connection to the LGBT community”), *intimacy* (e.g., “My LGBT identity allows me to be closer to my intimate partner”), and *social justice* (e.g., “My experience with my LGBT identity leads me to fight for the rights of others”) with five items per subscale using seven-point Likert-typed scales. Answer options range from ‘1 = *strongly disagree’* to ‘7 = *strongly agree*’ with an additional ‘0 = *does not apply*’ option added for this study (coded as missing).

The English version of the LGB-PIM (Riggle et al., 2014) has demonstrated good to excellent internal consistencies (subscale ranges: α = 0.82–0.95; Riggle et al., 2014; Rostosky et al., 2018) and test-retest reliabilities (subscale ranges: *r* = 0.54–0.87; Riggle et al., 2014), as well as convergent and incremental validities with/over other (sexual minority) identity and minority stress measures (Riggle et al., 2014). For the German-speaking survey, we used an unpublished German translation of the LGB-PIM by one of the authors (MS; no psychometric information available) that was created using the parallel-blind-technique (Behling & Law, 2000). Internal consistencies (coefficient α) were similar to English validation studies and good to excellent (self-awareness = 0.80, authenticity = 0.83, community = 0.91, intimacy = 0.83, social justice = 0.83). Subscale scores were formed by averaging across available item scores (if at least 80% of items were answered), with higher scores indicating higher levels of the respective positive identity dimension.

As to nomenclature regarding different conceptualizations of intimacy in the broader literature and in the LGB-PIM, we retained the original scale name of the LGB-PIM (i.e., “intimacy”) in methods and results sections. In the discussion, however, we use the phrase “capacity for intimacy” when referring to intimacy as assessed by the LGB-PIM.

#### Perceived Relationship Quality

Perceived relationship quality was assessed using the Perceived Relationship Quality Components Inventory (PRQC; Fletcher et al., 2000) that assesses six dimensions of perceived relationship quality with a current partner using three items respectively: *Satisfaction* (e.g., “How satisfied are you with your relationship?”), *commitment* (e.g., “How committed are you to your relationship?”), *intimacy* (e.g., “How intimate is your relationship?”), *trust* (e.g., “How much do you trust your partner?”), *passion* (e.g., “How passionate is your relationship?”), and *love* (e.g., “How much do you love your partner?”). Answers were recorded using a seven-point Likert-typed scale ranging from ‘1 = *not at all*’ to ‘7 = *extremely*’. The German translation for the PRQC was taken from an ongoing project by several co-authors (https://osf.io/rz3bt) investigating the factorial structure and validation of the PRQC in German-speaking countries. As data collection is still ongoing, psychometric information regarding the German version is currently unavailable.

Participants in polyamorous relationships were asked to answer with respect to the partner they spent the most time with. For the current study, we formed an overall score (if at least 80% of items were answered) of relationship quality by averaging across all answered items (higher scores indicate higher relationship quality). The formation of such a score is justified based on prior research (Fletcher et al., 2000). Subscale scores used in supplementary analyses (see OSF-Supplement S2) were formed by averaging across items (at least 80% of items answered) for each of the six subscales. Internal consistencies (coefficient α) in this study were excellent for the full score (0.93) and acceptable to excellent on a subscale-level (satisfaction: 0.95, commitment: 0.72, intimacy: 0.89, trust: 0.85, passion: 0.93, love: 0.69).

#### Psychological Distress

Psychological distress was assessed using the 21-item version of the Depression Anxiety and Stress Scales (DASS-21; Lovibond & Lovibond 1995; German version: Nilges & Essau 2015). The DASS-21 assesses depression (e.g., “I felt that I had nothing to look forward to”), anxiety (e.g., “I felt scared without any good reason”), and stress (e.g., “I found it hard to wind down”) experienced in the past week with seven items each using a 4-point Likert-typed scale. Answer options range from ‘0 = *Did not apply to me at all*’ to ‘3 = *Applied to me very much, or most of the time*’. Higher scores indicate higher distress. For the current study, we calculated an overall score of general psychological distress by averaging across items (at least 80% of items answered) by averaging across all available items (α = 0.96).

#### Sociodemographic Covariates

The following sociodemographic characteristics were used as covariates in all analyses: Country of residence, age (in years), sexual orientation, gender identity, and relationship length (in months). Sexual orientation (self-definition) was coded into three categories: Lesbian/gay, bi-/plurisexual, and queer/other. Gender identity was also coded into three categories: Cis-male (i.e., sex assigned at birth and current gender identity are male), cis-female, and gender minority (i.e., the current gender identity is different from the sex assigned at birth and/or the participant indicated another gender identity than male or female).

The complete codebook alongside the formation of analytical categories for sexual orientation, gender identity, and education levels (used for descriptive purposes only), is reported in the preregistration (https://osf.io/h4dnt/).

### Analytic Strategy

#### Confirmatory Factor Analysis of the LGB-PIM

For Hypothesis 1, we assessed the construct validity of the German version of the LGB-PIM using confirmatory factor analysis (CFA). Because we lacked an independent sample to test our final model in case of respecifications, we used a split-sample/cross-validation approach (Brown, 2015). To do so, we split the sample (stratified by country) into a test (*n* = 343) and a validation sample (*n* = 228). We opted for an unequal split (60:40) to allocate more statistical power to the test sample (where possible model re-specification would occur), while still preserving enough statistical power for a CFA in the validation sample.

We assumed the same item-factor structure as the original LGB-PIM, as well as correlated factors, uncorrelated error terms, and no item cross-loadings. We scaled latent variables by fixing their variance to 1 and used diagonally weighted least squares estimation with means and variances adjusted (WLSMV), treating items as ordinal-scaled (Li, 2016).

For all analyses, we assumed the following cut-offs for acceptable model fit, based on fit measures obtained from the original validation of the LGB-PIM (Riggle et al., 2014; SRMR = 0.07; RMSEA = 0.06; CFI = 0.91) and well-established recommendations: RMSEA (robust) and SRMR < 0.08 (Browne & Cudeck, 1993; Hu & Bentler, 1999); CLI and TFI (both robust) > 0.90 (Bentler, 1990). We considered standardized factor loadings to be salient if they exceeded 0.50. Analytic strategy and CFA results are outlined in detail in Supplement S3.

#### Regression Analyses

For Hypothesis 2, we examined the association between the positive identity aspects and relationship quality using hierarchical multiple regression. We regressed overall relationship quality on a set of predictors in the following steps: In Step 1, we added the five positive minority identity aspects. In Step 2, we added sociodemographic covariates, namely country of residence (Germany [reference category] vs. Austria vs. Switzerland), age (in years), sexual orientation (lesbian/gay [reference] vs. bi-/plurisexual vs. queer/other), gender identity (cis-male [reference] vs. cis-female vs. gender minority), and relationship length (in months). In Step 3 we added psychological distress as a conceptual covariate, as we were interested in incremental associations of positive minority identity aspects with relationship quality above those explained by psychological distress.

Continuous predictors were mean-centered prior to analysis, which allowed for the intercept in the unstandardized regression model to be interpreted as the expected relationship quality score for a participant with sample mean levels on all continuous variables and belonging to the reference category of categorical variables (i.e., living in Germany, lesbian/gay self-identification, cis-male gender identity). Standardized coefficients (β) are reported for conventional reasons and based on mean-centered variables divided by their standard deviation. Categorical (and dummy-coded) variables were not standardized. Semi-partial correlations (*r*_sp_) for regression coefficients were derived from respective *t*-statistics and *R*^2^-values and used for evaluation of absolute and relative effect strength (Aloe & Thompson, 2013).

#### Inference Criteria

Statistical significance was assumed at *p* < .05 (two-tailed). Effect strength was interpreted based on well-established cut-offs (Cohen, 1988) equivalent to *r* > |0.10|, |0.30|, |0.50| for lower thresholds of small, medium, and large effects respectively. In regression models, a variance inflation factor (VIF) > 4 (i.e., an increase in the predictor’s standard error by two compared to a model with zero correlations to other predictors) was deemed indicative of multicollinearity.

### Sensitivity Analyses

We additionally ran our regression models four times to rule out statistical and conceptual artifacts: (1) using LGB-PIM factor scores obtained from the CFA, (2) removing influential cases (Cook’s distance > 1; Cook & Weisberg 1982, and, in a more conservative analysis, Cook’s distance > 4/*N*; Bollen & Jackman 1990), (3) obtaining simple non-parametric bootstrapped confidence intervals around the coefficient estimates (5,000 samples) due to the non-normality of the data (not preregistered), and (4) using couple gender (mixed-gender [reference category] vs. same-gender vs. gender minority couple) instead of sexual orientation as a predictor. This was done to control for the perceived “majority status” (i.e., mixed-gender) of the relationship (Mark et al., 2020), which might influence the association between positive minority identity aspects and relationship quality. This predictor was not included in the main model because of collinearity concerns with sexual orientation.

## Results

### Descriptive Statistics and Bivariate Correlations

Descriptive statistics are reported in Tables 1 and 2. Overall, the sample reported low levels of psychological distress, as well as high levels of positive minority identity aspects and perceived relationship quality. Across countries, participants significantly differed regarding self-awareness, authenticity, and community (albeit to a small degree; η^2^ = 0.01 to 0.03), but not regarding psychological distress, intimacy, social justice, and relationship quality.

Bivariate correlations for all continuous variables are reported in Table 3. All five positive minority identity aspects showed significant, small to moderate associations with relationship quality (*r* = 0.12 for social justice to *r* = 0.35 for intimacy). The positive minority identity aspects, excepting self-awareness (*r* = − 0.04) and social justice (*r* = 0.08), also showed significant negative small to moderate associations with psychological distress (*r* = − 0.15 for intimacy to *r* = − 0.33 for authenticity).

**Table 3:** Pairwise Correlations and Internal Consistencies for Study Variables

Variable	1	2	3	4	5	6	7	8	9
1. Self-Awareness	0.80								
2. Authenticity	0.20***	0.83							
3. Community	0.24***	0.28***	0.91						
4. Intimacy	0.42***	0.29***	0.27***	0.83					
5. Social Justice	0.47***	0.12**	0.29***	0.34***	0.83				
6. Age	0.08	0.21***	0.02	0.05	− 0.07	–			
7. Relationship Length	0.05	0.14**	0.03	− 0.02	< |0.01|	0.66***	–		
8. Psychological Distress	− 0.04	− 0.33***	− 0.16***	− 0.15***	0.08	− 0.27***	− 0.15***	0.96	
9. Relationship Quality	0.14***	0.20***	0.15***	0.35***	0.12**	− 0.12**	− 0.05	− 0.16***	0.93

*Note*. Range bivariate *N* = 459–571. Coefficient alpha for scale scores is presented on the diagonal

*** *p* < .001, ** *p* < .01 (two-tailed)

### CFA of the German LGB-PIM

Details of model building and full results for the CFA are reported in OSF-Supplement S3. Global model fit was deemed acceptable in the test sample without further respecifications (*n* = 343; RMSEA = 0.07, SRMR = 0.08 [0.076], CFI = 0.97, TLI = 0.96), and subsequently in the full sample (*N* = 571; RMSEA = 0.07, SRMR = 0.07, CFI = 0.97, TLI = 0.96).

### Regression Analyses

#### Main Analysis

Results of the hierarchical multiple regression analysis are reported in Table 4. In Step 1, relationship quality was regressed on all five positive minority identity aspects (adj. *R*^2^ = 0.13). Only intimacy was significantly and positively associated with relationship quality (*r*_sp_ = 0.30), whereas all other positive minority identity aspects showed no significant associations. In Step 2, we entered sociodemographic covariates (i.e., country, age, sexual orientation, gender identity, relationship length) into the model (adj. *R*^2^ = 0.20). Intimacy remained a significant positive predictor of relationship quality (*r*_sp_ = 0.28), as were age (*r*_sp_ = − 0.19) and cis-female (vs. cis-male) gender (*r*_sp_ = 0.13). In Step 3, we entered psychological distress into the model (adj. *R*^2^ = 0.21), which was not significantly associated with relationship quality. Intimacy remained a significant predictor of relationship quality (*r*_sp_ = 0.28), even when controlling for sociodemographic characteristics and psychological distress. None of the other positive minority identity aspects where meaningfully associated with relationship quality in final models. The maximum VIF was 2.04, thus, multicollinearity was not considered to impact our results.

**Table 4:** Results from Main Hierarchical Regression Analysis Regressing Relationship Quality on Positive Minority Identity Aspects, Sociodemographic Characteristics, and Psychological Distress

Term	*b* (SE)	β	*t*	*p*	*r*_sp_
Step 1					
Intercept	6.00 (0.04)	–	158.38	**< 0.001**	
Self-Awareness	-0.04 (0.05)	-0.05 [-0.16; 0.06]	-0.91	0.362	− 0.04
Authenticity	0.06 (0.05)	0.07 [-0.03; 0.17]	1.31	0.190	0.06
Community	0.03 (0.03)	0.04 [-0.06; 0.14]	0.85	0.396	0.04
Intimacy	0.21 (0.03)	0.35 [0.24; 0.46]	6.40	**< 0.001**	0.30
Social Justice	0.01 (0.05)	0.01 [-0.10; 0.11]	0.16	0.876	0.01
Step 2					
Intercept	5.84 (0.10)	–	57.25	**< 0.001**	
Self-Awareness	> -0.01 (0.05)	< 0.01 [-0.11; 0.11]	-0.02	0.987	> − 0.01
Authenticity	0.06 (0.05)	0.06 [-0.04; 0.16]	1.23	0.220	0.06
Community	0.03 (0.03)	0.05 [-0.05; 0.14]	0.92	0.356	0.04
Intimacy	0.20 (0.03)	0.34 [0.23; 0.44]	6.22	**< 0.001**	0.28
Social Justice	> -0.01 (0.05)	-0.01 [-0.11; 0.10]	-0.10	0.924	> − 0.01
Germany vs. Austria	0.07 (0.09)	0.09 [-0.12; 0.30]	0.81	0.419	0.04
Germany vs. Switzerland	0.08 (0.11)	0.10 [-0.15; 0.36]	0.79	0.430	0.04
Age (Years)	-0.02 (< 0.01)	-0.26 [-0.39; -0.14]	-4.12	**< 0.001**	− 0.19
Lesbian/Gay vs. Bi-/Plurisexual	0.06 (0.10)	0.07 [-0.17; 0.30]	0.58	0.563	0.03
Lesbian/Gay vs. Queer/Other	-0.16 (0.12)	-0.20 [-0.50; 0.10]	-1.32	0.188	− 0.06
Cis-Male vs. Cis-Female	0.30 (0.11)	0.37 [0.11; 0.63]	2.83	**0.005**	0.13
Cis-Male vs. Gender Minority	-0.03 (0.13)	-0.03 [-0.34; 0.27]	-0.20	0.839	− 0.01
Relationship Length (Months)	< 0.01 (< 0.01)	0.11 [-0.01; 0.23]	1.79	0.074	0.08
Step 3					
Intercept	5.82 (0.10)	–	56.72	**< 0.001**	
Self-Awareness	> -0.01 (0.05)	> -0.01 [-0.12; 0.11]	-0.09	0.930	> − 0.01
Authenticity	0.04 (0.05)	0.04 [-0.06; 0.15]	0.83	0.404	0.04
Community	0.02 (0.03)	0.03 [-0.07; 0.13]	0.64	0.525	0.03
Intimacy	0.20 (0.03)	0.33 [0.23; 0.44]	6.21	**< 0.001**	0.28
Social Justice	0.01 (0.05)	0.01 [-0.10; 0.11]	0.12	0.903	0.01
Germany vs. Austria	0.07 (0.09)	0.09 [-0.12; 0.30]	0.81	0.419	0.04
Germany vs. Switzerland	0.08 (0.11)	0.10 [-0.16; 0.35]	0.74	0.461	0.03
Age (Years)	-0.02 (< 0.01)	-0.28 [-0.40; -0.15]	-4.35	**< 0.001**	− 0.19
Lesbian/Gay vs. Bi-/Plurisexual	0.07 (0.10)	0.09 [-0.15; 0.32]	0.73	0.465	0.03
Lesbian/Gay vs. Queer/Other	-0.16 (0.12)	-0.19 [-0.49; 0.10]	-1.28	0.202	− 0.06
Cis-Male vs. Cis-Female	0.31 (0.11)	0.39 [0.13; 0.64]	2.95	**0.003**	0.13
Cis-Male vs. Gender Minority	0.02 (0.13)	0.03 [-0.28; 0.34]	0.18	0.859	0.01
Relationship Length (Months)	< 0.01 (< 0.01)	0.11 [-0.01; 0.23]	1.80	0.073	0.08
Psychological Distress	-0.12 (0.07)	-0.10 [-0.2; 0.01]	-1.87	0.063	− 0.08

*Note*. *N* = 396. *b* (SE) = unstandardized predictor and standard error. β = standardized coefficient with 95% confidence interval in square brackets, *t* = *t*-statistic, *r*_sp_ = semi-partial correlation. Categorical predictors were not standardized. Significant (*p* < .05) estimates are in bold

Step 1: *R*^2^ = 0.14, adj. *R*^2^ = 0.13, F(5, 390) = 12.94, *p* < 0.001, max. VIF = 1.43.

Step 2: *R*^2^ = 0.23, adj. *R*^2^ = 0.20, F(13, 382) = 8.79, *p* < 0.001, max. VIF = 2.01.

Step 3: *R*^2^ = 0.24, adj. *R*^2^ = 0.21, F(14, 381) = 8.46, *p* < 0.001, max. VIF = 2.04.

#### Supplementary Analysis: Subdimensions of Relationship Quality

In a series of supplementary regression analyses, we explored differential patterns of associations between positive minority identity aspects and the six subdimensions of relationship quality according to the PRQC (Fletcher et al., 2000; OSF-Supplement S2). Again, intimacy, but no other positive minority identity aspect, was significantly and positively associated with five out of six subdimensions of relationship quality (i.e., satisfaction, commitment, intimacy, passion, and love) in final models (range *r*_sp_ = 0.21–0.27). However, intimacy (and every other positive minority identity aspect) was unrelated to the subdimension trust in the final model.

#### Sensitivity Analyses

Our main results (i.e., intimacy was positively associated with relationship quality) remained robust in several further sensitivity analyses. These included using factor scores (Supplement S4), removing outliers as defined by two different thresholds of the Cook’s distance (Supplement S5), obtaining bootstrapped confidence intervals (Supplement S6), and using couple gender instead of sexual orientation as a predictor (Supplement S7). Intimacy was significantly and positively associated with relationship quality in all analyses (range *r*_sp_ = 0.24–0.27). Again, all other positive minority identity aspects showed only trivial and non-significant associations with relationship quality. Bootstrapped results only trivially differed from not-bootstrapped results.

## Discussion

The intimate relationships of sexual minorities are receiving growing interest (Meuwly & Randall, 2019; Rostosky & Riggle, 2017), but investigations often adopt a deficit-oriented and US-centered perspective. Therefore, we explored associations between five positive minority identity aspects (self-awareness, authenticity, community, capacity for intimacy, and social justice; Riggle et al., 2014) and relationship quality in a sample of sexual minorities from German-speaking countries (i.e., Austria, Germany, and Switzerland).

Prior to conducting our main analyses, we hypothesized that the LGB-PIM (Riggle et al., 2014) would show acceptable model fit in our German-speaking sample. Model fit was deemed acceptable in our analyses. We further expected that all five positive minority identity aspects would show positive associations with relationship quality. This hypothesis was only partially supported. All five positive minority identity aspects displayed small to moderate bivariate associations with relationship quality, but only capacity for intimacy was significantly associated with relationship quality in higher-order models. These results remained robust in several sensitivity analyses and generalized across subdimensions of relationship quality (excepting trust).

The positive association between capacity for intimacy and relationship quality in our study is in line with theory (Fletcher et al., 2000; Reis & Shaver, 1988). At a first glance, the results of the current study might thus simply be a generalization of findings from previous research that has been conducted with heterosexual individuals. However, the operationalization of intimacy as assessed by the LGB-PIM (Riggle et al., 2014) warrants a closer look: In the LGB-PIM, intimacy is conceptualized as believing that one’s sexual (or gender) identity “enhances one’s capacity for intimacy and sexual freedom” (Riggle et al., 2014, p. 404). The (US-based) qualitative works that served as the basis for the LGB-PIM elucidate this operationalization further: Participants reported an increased freedom to explore different expressions of sexuality and relationships due to freedom from gendered roles (Riggle et al., 2008; Rostosky et al., 2010). Our findings and their implications should thus be viewed in the light of a broader social and legal climate: In German-speaking countries, consensual same-gender sexual acts between adults were criminalized up to 1942 in Switzerland, 1968/1969 in Germany (East/West) and 1971 in Austria (ILGA World et al., 2020). Further, as noted in the introduction, full marriage equality in these countries is only a very recent phenomenon (2017–2021), whereas previous institutions were not legally equal to mixed-gender marriages (ILGA World et al., 2020).

While Western societies have become more accepting towards non-heterosexuality (Smith et al., 2014), large-scale studies document the pervasive and insidious nature of discrimination and marginalization against sexual minorities and their relationships in these societies to date (European Union Agency for Fundamental Rights, 2020). In addition, studies on inclusive (sexual) education further highlight critical gaps in curricula and the adverse ramifications of failure to address diverse sexual orientations and gender identities in an inclusive manner, even for contemporary youth (i.e., in studies from 2014 onwards; Epps et al., 2021). In a recent EU-wide large-scale survey on sexual and gender minorities, 71% (Austria) to 77% (Germany) of participants reported that issues relating to diverse sexual orientations, gender identities, or sex characteristics were not addressed during their school education at any point, and further 4% (both countries) reported that these issues were only addressed in a negative way (European Union Agency for Fundamental Rights, 2020).

Thus, participants in our study may have grown up exploring their sexuality and intimate relationships in a sociolegal climate where diverse sexual orientations (and consequently relationships) were not legalized and not spoken about at best and contested at worst. Previous studies on the detrimental impact of minority stress on relational outcomes underscore how the ramifications of societal marginalization influence relationship functioning in sexual minority populations (Cao et al., 2017; Doyle & Molix, 2015). Against this background, the positive association between higher reports of an increased capacity for intimacy and relationship quality found in our study is particularly noteworthy. Specifically, it highlights the importance of overcoming heteronormative notions of intimate relationships and sexuality imposed by societal and legal norms for relational well-being and – by extension – inclusive education, counselling, and public discourse.

Patterns of associations between other positive minority identity aspects and relationship quality were less clear in our study. Excepting capacity for intimacy, we found significant but small bivariate associations between all identity aspects and relationship quality that were not significant in higher-order models. It is conceivable that the low variation with respect to both outcome and predictor variables in combination with a simultaneous consideration in higher-order models might have led to lower effect estimates; see the Limitations below for more information. Future studies might wish to assess the model fit of one (or more) higher-order factors (Sommantico et al., 2019, 2020) or administer only relevant subscales of the LGB-PIM (Riggle et al., 2014).

Conceptually, positive minority identity aspects might not be associated with how the relationship or the partner is *perceived by the participant*, as operationalized by the PRQC (Fletcher et al., 2000). Rather, positive minority identity aspects might be associated with how participants (or the relationship) are *perceived by their partner*: Heightened self-awareness or valuing social justice might be related to being perceived as a more considerate, authentic, and empathic partner, but might be unrelated to one’s own perception of the relationship (or the partner). Dyadic data collection efforts, ideally applying a longitudinal design, could disentangle actor (i.e., associations between Partner A’s predictor and their outcome) and partner (i.e., associations between Partner A’s predictor and Partner B’s outcome) effects further, for example by using the actor-partner interdependence model (Kenny & Ledermann, 2010). Researchers may wish to not only investigate hypotheses related to actor and partner effects, but also to dyadic effects (e.g., dissimilarity in levels of positive minority identity aspects between partners might contribute to relationship satisfaction).

Among our covariates, cis-female (compared to cis-male) gender and being in a same-gender couple (compared to being in a gender minority couple; supplementary) were positively associated with relationship quality, whilst sexual orientation (lesbian/gay compared to bi-/pansexual and compared to queer/other) was not. There is limited information on gender differences in relationship functioning in sexual minorities (Song et al., 2021). Some studies corroborate our findings regarding cis-female vs. cis-male gender differences (Guschlbauer et al., 2019; Sommantico et al., 2019, 2020), but contrasting and null evidence exist (Rice et al., 2020; Totenhagen et al., 2018). Evidence regarding disparities in relationship functioning in gender minority populations is even more limited (Marshall et al., 2020).

Regarding sexual orientation, our null findings are in line with previous non-significant results (Mark et al., 2015). To this end, we considered gender identity, sexual orientation, and couple gender merely as covariates in our study. Researchers interested in group differences are encouraged to model the complex interactions between these variables, which allow for a more meaningful picture. For example, researchers could examine intersections between individual’s identity that may impact their experiences (intersection between race or ethnicity, sexual orientation, and gender identity, as an example).

Age (but not relationship length) was negatively associated with relationship quality. Cross-sectional studies focusing on positive relationship functioning in sexual minorities yield mixed results for both variables. For age, there is evidence for negative (Sommantico et al., 2019, 2020), positive (Vale & Bisconti, 2021), no (Pepping et al., 2019), or differential (Totenhagen et al., 2018) associations. Similarly, for relationship length, there is evidence for no (Rice et al., 2020; Vale & Bisconti, 2021) or differential (Totenhagen et al., 2018) associations. This is not surprising, as a recent meta-analysis with longitudinal studies from the general (i.e., presumably mostly heterosexual) population found evidence for non-linear relations between relationship satisfaction and age and relationship length respectively (Bühler et al., 2021). Thus, our cross-sectional, between-person design (as well as evidence cited above) does not allow for an understanding of the longitudinal, within-person (and within-couple) effects of these variables on relationship quality (see Brauer et al., 2022, for an in-depth discussion).

Across countries, participants did not differ in their levels of self-reported relationship quality. This is noteworthy, as this represents one of the first tri-nation studies in German-speaking countries that assesses aspects of relationship functioning in sexual minorities. In Switzerland, where marriage was not legalized at time of data collection, participants even reported the highest levels of relationship quality (albeit not significantly). As data collection took place in the months leading up to the respective referendum (summer – fall 2021), Swiss participants might have been particularly attuned to positive aspects of their (contested) relationship and/or wanted to depict non-heterosexual relationships as particularly positive (see Limitations).

Psychological distress was significantly related to relationship quality in a bivariate analysis (*r* = − 0.16) and some sensitivity (using factor scores), and supplementary analyses (satisfaction dimension of the PRQC [Fletcher et al., 2000]), but did not show meaningful associations in our main regression model. This is surprising, as mental health is robustly linked to relationship functioning (Braithwaite & Holt-Lunstad, 2017; Proulx et al., 2007). Studies on sexual minorities have found associations (Feinstein et al., 2019; Frost & Meyer, 2009; Guschlbauer et al., 2019; Haas & Lannutti, 2021; Liang & Huang, 2021; Vale & Bisconti, 2021) between relationship quality (e.g., satisfaction) and negative mental health outcomes (e.g., depression) in small to moderate ranges (*r*s = − 0.17 to − 0.41). Our estimate borders this lower threshold. Different operationalizations, sampling strategies, and sample characteristics (e.g., low variability in our sample leading to effect underestimation) might contribute to these discrepant findings.

### Limitations and Future Research

First, relating to sampling biases, our participants had to have some form of affiliation with the LGBTIQA + community, as community organizations served as our primary multipliers for study dissemination. This is a ubiquitous limitation when relying on convenience samples from this population (Meyer & Wilson, 2009), as limited resources do not allow for any non-targeted sampling approaches due to the low base rate of non-heterosexual orientations or non-cisgender identities. Whilst some evidence points to higher mental health burden in convenience than population-based samples (Hottes et al., 2016), we do not know of any study contrasting relational outcomes in convenience vs. population-based samples, particularly so in a German-speaking context. This limitation emphasizes the need for population-based data on sexual and gender minorities in German-speaking countries (Bränström et al., 2019), focusing not only on mental health but also on relational outcomes.

Second, our sample exhibited little variation in some positive minority identity aspects and relationship quality. Thus, findings from bi- and multivariate analyses should be regarded as lower, rather than an upper, thresholds. High levels of relationship quality are a well-known limitation in relationship research using convenience samples (Fowers et al., 2001; Zemp et al., 2017), which certainly applies to our study as well. Since studies on relationship functioning are few for sexual and gender minorities in German-speaking countries, it is conceivable that this further introduced a social desirability bias, with participants attempting to depict their relationships in a particularly positive light.

Third, the broad term “LGBT identity” is used in the LGB-PIM items to refer to participants’ identities. While this reflects a conscious choice by the scale creators with many administrative advantages (Riggle et al., 2014), it could give rise to differential item functioning for participants who have been marginalized within the LGBT community and thus may find many positive aspects related to their own sexual orientation or gender identity, but not related to a collective LGBT identity. Future studies could investigate this notion further by administering items tailored to participants’ self-reported identity.

Fourth, we included a measure of psychological distress to assess the incremental association between positive minority identity aspects and relationship quality beyond mental health. As associations of positive minority identity aspects with positive mental health outcomes (e.g., well-being) are usually stronger than with negative mental health outcomes (e.g., psychological distress; Riggle et al., 2014; Rostosky et al., 2018), an inclusion of a positive mental health measure might be fruitful in future research.

Fifth, no causality regarding associations can be derived from our cross-sectional data. Related, data collection took place amidst the COVID-19 pandemic in summer and early fall of 2021. While this limitation pertains to all psychological research from 2020 onwards, evidence suggests that the pandemic brought about unique stressors for sexual and gender minorities (Salerno et al., 2020) that might have differentially impacted reports of mental health (which, however, was generally high in our sample) and relationship quality.

## Conclusions

A sizeable body of evidence documents the detrimental impact of minority stress on relational outcomes in sexual minorities (Rostosky & Riggle, 2017), but positive minority identity aspects remain mostly overlooked. In this tri-nation study of sexual minorities living in a German-speaking country (Austria, Germany, or Switzerland) we found that a greater self-reported capacity for intimacy because of one’s non-heterosexual identity was related to higher self-reported relationship quality. Other positive minority identity aspects seemed to contribute little to relationship quality when considered simultaneously. Mental health practitioners working with sexual minority individuals, or couples, may wish to explore their client’s sexual identities beyond heteronormative assumptions, as this can strengthen the capacity for intimacy and relational well-being in sexual minorities. Promoting inclusive education beyond heteronormative assumptions of romantic relationships and sexuality may aid sexual minority youth in a critical developmental period and contribute to positive relationship functioning in adulthood (Mustanski et al., 2014). Our study highlights the importance of positive psychological research to elucidate drivers of individual and relational well-being in sexual minorities.

## Data Availability

Data, R code, codebook, and supplementary materials are available at: https://osf.io/94k8x/. The manuscript was posted as a preprint to: https://psyarxiv.com/4k97w.
